# Enhancing of cerebral Abeta clearance by modulation of ABC transporter expression: a review of experimental approaches

**DOI:** 10.3389/fnagi.2024.1368200

**Published:** 2024-05-30

**Authors:** David A. Loeffler

**Affiliations:** Department of Neurology, Beaumont Research Institute, Corewell Health, Royal Oak, MI, United States

**Keywords:** ABC transporters, Abeta, Alzheimer’s, cerebral clearance, experimental approaches

## Abstract

Clearance of amyloid-beta (Aβ) from the brain is impaired in both early-onset and late-onset Alzheimer’s disease (AD). Mechanisms for clearing cerebral Aβ include proteolytic degradation, antibody-mediated clearance, blood brain barrier and blood cerebrospinal fluid barrier efflux, glymphatic drainage, and perivascular drainage. ATP-binding cassette (ABC) transporters are membrane efflux pumps driven by ATP hydrolysis. Their functions include maintenance of brain homeostasis by removing toxic peptides and compounds, and transport of bioactive molecules including cholesterol. Some ABC transporters contribute to lowering of cerebral Aβ. Mechanisms suggested for ABC transporter-mediated lowering of brain Aβ, in addition to exporting of Aβ across the blood brain and blood cerebrospinal fluid barriers, include apolipoprotein E lipidation, microglial activation, decreased amyloidogenic processing of amyloid precursor protein, and restricting the entrance of Aβ into the brain. The ABC transporter superfamily in humans includes 49 proteins, eight of which have been suggested to reduce cerebral Aβ levels. This review discusses experimental approaches for increasing the expression of these ABC transporters, clinical applications of these approaches, changes in the expression and/or activity of these transporters in AD and transgenic mouse models of AD, and findings in the few clinical trials which have examined the effects of these approaches in patients with AD or mild cognitive impairment. The possibility that therapeutic upregulation of ABC transporters which promote clearance of cerebral Aβ may slow the clinical progression of AD merits further consideration.

## 1 Introduction

In 2023 approximately 6.7 million Americans age 65 and older were living with Alzheimer’s disease (AD)–related dementia (Alzheimer’s Association, 2023). Worldwide, AD has been suggested to account for 60–70% of the approximately 55 million cases of dementia (Mayo Clinic, 2023a). AD’s hallmark pathological findings are amyloid beta protein (Aβ)–containing senile plaques (SPs) and tau protein–containing neurofibrillary tangles. The etiology of familial (early onset) AD (AD-related dementia developing before age 65) is thought to be due to mutations in the amyloid precursor protein (APP), Presenilin 1 (*PSEN1*), and Presenilin 2 (*PSEN2*) genes (Petit et al., 2022). The etiology of late onset AD (LOAD; AD-related dementia developing at age 65 or later) remains unclear, with multiple factors including gene mutations, environmental toxins, and infectious agents suggested as possible contributing factors to its development or progression (Balin and Hudson, 2014; Chen et al., 2021; Bellenguez et al., 2022). Since publication of the amyloid hypothesis (Hardy and Allsop, 1991; Hardy and Higgins, 1992), which suggested that AD neuropathology was initiated by deposition of insoluble Aβ as SPs, therapeutic approaches have focused primarily on lowering of cerebral Aβ. These approaches have included Aβ vaccination (Gilman et al., 2005), Aβ aggregation inhibitors (Aisen et al., 2011), β-secretase inhibitors (Moussa-Pacha et al., 2020), γ-secretase modulators and inhibitors (Green et al., 2009; Imbimbo et al., 2011), and anti-Aβ monoclonal antibodies (Doody et al., 2014; Panza et al., 2014; Salloway et al., 2014). These approaches failed to slow the clinical progression of AD in large-scale clinical trials until recent trials with two monoclonal anti-Aβ antibodies, Lecanemab and Donanemab. Lecanemab slowed the progression of early stage AD by 27% and Donanemab slowed it by 32% (Sims et al., 2023; van Dyck et al., 2023). Whether these effects are clinically meaningful has been questioned (Alzforum, 2021; Prillaman, 2022).

The findings in the recent clinical trials with Lecanemab and Donanemab support the amyloid hypothesis. However, despite marked lowering of brain levels of PET-detectable Aβ by both antibodies, AD’s clinical progression continues, albeit more slowly than in placebo-treated patients. This suggests that more aggressive targeting of Aβ, including its soluble conformations, might further slow AD clinical progression. Alternatively, targeting of other pathology-related mechanisms suggested by the amyloid hypothesis to occur downstream of Aβ aggregation such as tau phosphorylation and aggregation, oxidative stress, and/or inflammation may be required to achieve this goal.

The literature includes descriptions of many experimental approaches for reducing brain levels of Aβ. Experimental approaches for increasing proteolytic degradation and antibody-mediated clearance of Aβ were recently reviewed by this author (Loeffler, 2023a,b). Additional mechanisms through which Aβ is cleared from the brain include its efflux across the blood brain barrier (BBB) (Qosa et al., 2014; Versele et al., 2022) and blood cerebrospinal fluid barrier (BCSFB) (Crossgrove et al., 2005; Shen et al., 2020), glymphatic drainage (Iliff et al., 2012; Li et al., 2022), and perivascular drainage (Bell et al., 2007; Zhang et al., 2021). ATP-binding cassette transporters, commonly known as ABC transporters, are membrane efflux pumps driven by hydrolysis of ATP (ElAli and Hermann, 2011; ElAli and Rivest, 2013; Erdő and Krajcsi, 2019). Some ABC transporters are present on the BBB and BCSFB (Morris et al., 2017). The present review discusses ABC transporters which have been suggested to promote lowering of cerebral Aβ, changes in their expression and/or activity in AD brain, experimental approaches which have been used to increase their expression, and findings in clinical trials which have explored the effects of these approaches in patients with AD or mild cognitive impairment (MCI).

**FIGURE 1:**
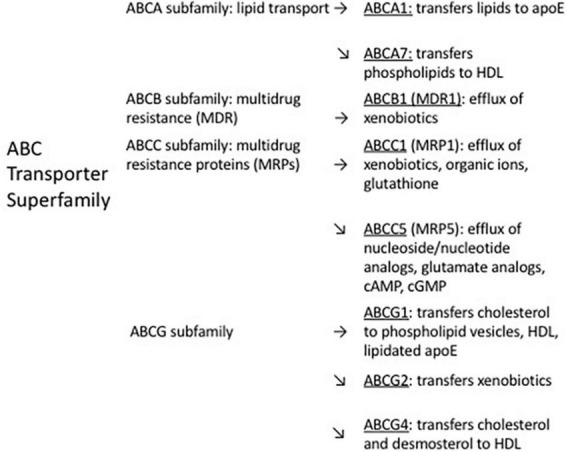
Distribution within ABC transporter subfamilies, and main efflux functions, of ABC transporters suggested to contribute to efflux of cerebral Aβ.

The main functions of ABC transporters are maintenance of normal brain homeostasis (Jha et al., 2019) and transport of bioactive molecules (Leslie et al., 2005; ElAli and Rivest, 2013; Moore et al., 2023). The ABC transporter superfamily in humans is comprised of 49 proteins, divided into seven subfamilies (Pahnke et al., 2014). These subfamilies have different kinetics for exporting their substrates (Krohn et al., 2011). Much of the early research on ABC transporters focused on their roles in tumor drug resistance (Biedler and Riehm, 1970; Chen et al., 1986; Tan et al., 2000; Szakács et al., 2006). Among the ABC transporters which contribute to clearance of Aβ from the brain, ABCB1 and ABCA1 have been most extensively studied (ElAli and Rivest, 2013). Both are highly expressed on brain capillary endothelial cells. ABCB1 is present on the luminal side of the BBB (Roberts et al., 2008; Hermann and ElAli, 2012; Osgood et al., 2017) and on the apical side of the choroid plexus epithelial cells of the BCSFB (Morris et al., 2017), while ABCA1 is on the abluminal side of the BBB (Panzenboeck et al., 2002). Other ABC transporters suggested to influence cerebral Aβ levels (reviewed by Pahnke et al., 2014) include ABCA7 (Kim et al., 2013; Aikawa et al., 2018), ABCC1 (Krohn et al., 2011; Hofrichter et al., 2013), ABCC5 (Shubbar and Penny, 2020), ABCG1 (Zelcer et al., 2007), ABCG2 (Xiong et al., 2009; Do et al., 2012), and ABCG4 (Do et al., 2012). Reports were found for the expression of each of these transporters on the BBB (ABCB1: Roberts et al., 2008; ABCA1: Panzenboeck et al., 2002; ABCA7: Dib et al., 2021; ABCC1: Bernstein et al., 2014; ABCC5: Jansen et al., 2015; ABCG1: Kober et al., 2017; ABCG2: Schulz et al., 2023; ABCG4: Dodacki et al., 2017) and on the BCSFB (or choroid plexus) (ABCB1: Møllgård et al., 2017; ABCA1: Liddelow et al., 2012; ABCA7: Tijms et al., 2024; ABCC1: Møllgård et al., 2017; ABCC5: Strazielle and Ghersi-Egea, 2015; ABCG1: Liddelow et al., 2012; ABCG2: Møllgård et al., 2017; ABCG4: Fujiyoshi et al., 2007; Liddelow et al., 2012; Matsumoto et al., 2015). Figure 1 shows the subfamily distribution and main efflux functions of ABC transporters suggested in the literature to participate in Aβ clearance, and Table 1 shows the mechanisms of regulation of these transporters and their alterations in expression in AD brain, if known.

**TABLE 1:** Main functions of ABC transporters involved in Aβ efflux.

ABC transporter	Functions	Regulation	Expression in AD brain
ABCA1	Transfer of cholesterol, other lipids to apoE	LXRs	Decreased
ABCA7	Transfer of phospholipids to HDL; mediates macrophage, microglial phagocytosis	SREBP2 pathway	Increased; gene variants are risk factors for LOAD
ABCB1 (p-gp)	Efflux of xenobiotic substances from cells; works with LRP1 to promote Aβ efflux across BBB	Nuclear receptors (RXR, PXR, PPAR, others), inflammation, oxidative stress, receptor tyrosine kinases, growth factors	Decreased
ABCC1	Efflux of xenobiotics, organic anions, glutathione	Notch1, transcription factors (GC elements, E-box elements)	Unclear; increased in AD mouse models
ABCC5	Efflux of nucleoside/nucleotide analogs, glutamate analogs, cAMP, cGMP	Forkhead box M1, dexamethasone, human chorionic gonadotropin	Unknown; activity decreased in vitro after exposure of endothelial cells to Ab42
ABCG1	Transfers cholesterol to HDL, phospholipid vesicles, lipidated apoE	LXR, PPAR activators, RXR agonists	Unknown; ABCG1 SNPs reported to be AD risk factors
ABCG2	Transport of xenobiotics	Inflammatory cytokines, dexamethasone, hypoxia, hormones, growth factors, gene amplification, epigenetic regulation	Conflicting reports
ABCG4	Efflux of cholesterol and desmosterol to HDL	LXRs	Increased in AD plaque-associated microglia

AD, Alzheimer’s disease; ApoE, apolipoprotein E; BBB, blood brain barrier; cAMP, cyclic adenosine monophosphate; cGMP, cyclic guanosine monophosphate; HDL, high density lipoproteins; LOAD, late onset Alzheimer’s disease; LRP1, low density lipoprotein receptor-related protein 1; LXR, liver X receptor; PPAR, peroxisome proliferator–activated receptor; PXR, pregnane X receptor; RXR, retinoid X receptor; SNP, single nucleotide polymorphism; SREBP2, sterol regulatory element-binding protein 2.

## 2 Materials and methods

Mechanisms of Aβ clearance from the brain were initially examined by a PubMed search of “Abeta clearance.” Some of the references that were found discussed the effects of various ABC transporters on Aβ clearance and the mechanisms, if known, by which the transporters promote this process. Further PubMed searches were performed using, as search terms, the ABC transporters suggested to be involved in ABC clearance, plus “Aβ clearance” and “experimental approaches.” The initial and subsequent literature searches identified published experimental approaches which have been used to increase the expression of these transporters. Clinical applications of the experimental approaches (including clinical trials and reports or reviews of their clinical use) were found through these PubMed searches and by searching ClinicalTrials.gov for the relevant ABC transporters and for the experimental approaches found to increase the expression of these transporters.

## 3 ABC transporters involved in clearance of cerebral Aβ

### 3.1 ABCA1

ABCA1 is expressed in the CNS by glia, neurons, and endothelial cells. It controls efflux of cholesterol, phospholipids, and other lipids from cells to systemic and brain apolipoprotein E (apoE) (Hirsch-Reinshagen et al., 2004; Do et al., 2011; ElAli and Rivest, 2013; Lewandowski et al., 2022). Its expression in mouse lateral ventricular choroid plexus was reported by Liddelow et al. (2012). Of relevance to AD, poor lipidation of apoE4 increases its instability, possibly leading to reduced clearance of Aβ, particularly neurotoxic Aβ oligomers (Tai et al., 2014; Lewandowski et al., 2022). The possibility that ABCA1 deficiency may promote increased aggregation of Aβ was suggested by a study which found that in the APP transgenic mouse model of AD, mice with only one copy of the *ABCA1* gene had increased brain levels of oligomeric Aβ (Lefterov et al., 2009). Transcription of the *ABCA1* gene is controlled by liver X receptors (LXRs) (Repa et al., 2000), which are present on glial cells and neurons (Jasmin et al., 2014). LXRs respond to elevated intracellular cholesterol by increasing the expression of genes which promote efflux of cholesterol and other lipids from cells (Valledor et al., 2004; Kalaany and Mangelsdorf, 2006; Wente et al., 2007). LXRs form complexes with retinoid X receptors (RXRs) (Heyman et al., 1992; de Urquiza et al., 2000) and these complexes bind to LXR response elements (Song et al., 1994; Willy et al., 1995). Experimental approaches which have been used to increase ABCA1 expression are shown in Table 2.

**TABLE 2:** Experimental approaches for increasing ABCA1 expression.

Experimental approach	References
ABCA1 overexpression^a^	Wahrle et al., 2008
Bexarotene^b^	Cramer et al., 2012; Balducci et al., 2015; Skerrett et al., 2015; Muñoz-Cabrera et al., 2019
Extra virgin olive oil^c^	Helal et al., 2013
LXR agonists^d^	Fukumoto et al., 2002; Koldamova et al., 2003; Sun et al., 2003; Burns et al., 2006; Donkin et al., 2010; Fitz et al., 2010; Loane et al., 2011; Vanmierlo et al., 2011
PPAR-α and PPAR-γ activation^e^	Chawla et al., 2001; Chinetti et al., 2001; Xia et al., 2005
Retinoic acid receptor agonists^f^	Costet et al., 2003
Verapamil^g^	Suzuki et al., 2004

^a^Overexpression of ABCA1 decreased cerebral Aβ burden and SPs in PDAPP transgenic mice.

^b^Bexarotene lowered cerebral Aβ in APP/PS1 and C57Bl/6 mice (Cramer et al., 2012) and a later study had similar findings in APP/PS1 mice (Skerrett et al., 2015). Bexarotene treatment lowered astrogliosis and reactive microglia, while increasing NeuN expression, in 3×Tg-AD mice (Muñoz-Cabrera et al., 2019). Conversely, Balducci et al., 2015, found no reduction in SPs in Bexarotene-treated TASTPM mice.

^c^Healthy human subjects consumed extra-virgin olive oil for 12 weeks. Plasma-derived HDL from these subjects increased *in vitro* cholesterol efflux from human monocyte-derived macrophages.

^d^Treatment of neuroblastoma cells with a LXR ligand increased their secretion of Aβ40 and Aβ 42 (Fukumoto et al., 2002). Exposure of primary neurons and glia from embryonic rat brain to LXR ligands increased their cholesterol efflux (Koldamova et al., 2003). Incubation of APP-expressing cells with LXR activators lowered their Aβ production independently of cellular lipid efflux (Sun et al., 2003). Treatment of wild-type mice with a LXR agonist increased plasma cholesterol and decreased cerebral Aβ (Burns et al., 2006). Administration of a LXR ligand to APP23 mice lowered cerebral Aβ, including SPs (Fitz et al., 2010), with similar results in APP/PS1 mice (Donkin et al., 2010). Pre- and post-injury treatment of C57Bl/6 mice undergoing traumatic brain injury (TBI) reduced their TBI-related increase in cerebral Aβ (Loane et al., 2011). Administration of a LXR activator to APPSLxPS1mut mice improved their memory functions without influencing cerebral Aβ levels (Vanmierlo et al., 2011).

^e^PPAR-γ treatment of tissue plasminogen activator-differentiated THP-1 macrophages increased cholesterol efflux (Chawla et al., 2001), and similar results were found for PPAR-α and PPAR-γ activation of human macrophages (Chinetti et al., 2001). Anthocyanin increased PPAR-γ-mediated cholesterol efflux from mouse peritoneal macrophages (Xia et al., 2005).

^f^Retinoic acid receptor activators increased ABCA1 expression and cholesterol efflux in mouse peritoneal macrophages, and ABCA1 expression in human monocytes/macrophages (Costet et al., 2003).

^g^The calcium channel blocker Verapamil increased ABCA1 expression and cholesterol efflux cAMP analog-treated RAW264 (mouse macrophage) cells (Suzuki et al., 2004). ABCA1, ATP binding cassette subfamily A member 1; LXR, liver X receptors; PPAR-α, peroxisome proliferator-activated receptor-α; PPAR-γ, peroxisome proliferator-activated receptor-γ.

Two of these approaches, the use of LXR agonists and the retinoid Bexarotene, merit further mention. LXR agonists may cause significant deleterious side effects (Xu et al., 2013) including increased levels of plasma triglycerides (Koldamova et al., 2010). Bexarotene, an RXR agonist (Gaunt et al., 2021), is an FDA-approved drug that stimulates both LXR and peroxisome proliferator-activated receptor gamma (PPAR-γ) pathways. Its administration to transgenic mouse models of AD produced conflicting results regarding its ability to reduce Aβ plaque numbers and slow cognitive decline (Cramer et al., 2012; Tai et al., 2014; Balducci et al., 2015; Skerrett et al., 2015; Muñoz-Cabrera et al., 2019; Ren et al., 2019). The differing results in these studies may have been due to differences in the rate of development or extent of Aβ pathology between the mouse models, or differing Bexarotene doses or timing of its administration. Bexarotene and its derivatives may lower Aβ concentrations in transgenic mouse models of AD via ABCA1- and ABCG1-mediated increase in apoE lipidation, although increased microglial phagocytosis and enzymatic degradation of Aβ has also been detected (Yuan et al., 2019).

Interestingly, the expression of ABCA1 in human neuroglioma cells was decreased by exposure to the pesticide dichlorodiphenyltrichloroethane (DDT) (Li G. et al., 2015), supporting the possibility of an association between AD and DDT exposure which was suggested in an earlier study (Richardson et al., 2014).

### 3.2 ABCA7

ABCA7 shares 54% sequence homology with ABCA1 (Kaminski et al., 2000). However, ABCA7 expression is regulated by a different mechanism than ABCA1 (Abe-Dohmae and Yokoyama, 2021), namely the sterol-responsive/regulatory element binding protein (SREBP) pathway, which upregulates expression of genes involved in cholesterol synthesis in response to cholesterol deficits (Iwamoto et al., 2006; Dib et al., 2021). Like ABCA1, the main function of ABCA7 may be to regulate lipid metabolism, although the main lipids exported by the two transporters differ (Tomioka et al., 2017; Aikawa et al., 2018): ABCA7 transfers phospholipids to high density lipoprotein (HDL) whereas ABCA1 transfers cholesterol (Dib et al., 2021). ABCA7 is expressed in the CNS by neurons, microglia, astrocytes, and endothelial cells (Kim et al., 2006; Zhang et al., 2014; Fu et al., 2016) and Tijms et al. (2024), in a cerebrospinal fluid (CSF) proteomics study, mentioned its expression in choroid plexus; Liddelow et al. (2012) also reported its detection on mouse choroid plexus. It mediates macrophage and microglial phagocytosis (Fu et al., 2016). *ABCA7* gene variants are risk factors for LOAD (Naj et al., 2011; Reitz et al., 2013; Steinberg et al., 2015; Efthymiou and Goate, 2017). In transgenic mouse models of AD, *ABCA7* knockout increases SP counts. There are conflicting reports regarding the mechanism responsible for this increase. Knockout of *ABCA7* in APP/PS1 mice resulted in increased production of Aβ due to elevated activity of β-site APP cleaving enzyme 1 (Sakae et al., 2016), similar to findings in an *in vitro* study (Satoh et al., 2015). However, knockout of *ABCA7* in J20 mice, which express both the Swedish (K670N/M671L) and the Indiana (V717F) APP mutations, produced no changes in APP processing; increased SP density in J20 mice after *ABCA7* knockout was suggested to be due to impaired phagocytic clearance of Aβ (Kim et al., 2013; Li H. et al., 2015). In an *in vitro* model of the BBB, reduction of ABCA7 expression induced by small interfering RNA decreased basolateral-to-apical transport of Aβ in mouse endothelial cells. ABCA1 expression was also decreased, and this may have contributed to the decrease in Aβ transport (Lamartinière et al., 2018). ABCA7 expression has been reported to be increased in AD brain, possibly as a compensatory mechanism for increased Aβ burden (Vasquez et al., 2013).

Experimental approaches to increase ABCA7 expression appear to have been limited to gene transfection (Chan et al., 2008) and statin-induced blocking of cholesterol synthesis (Tanaka et al., 2010).

### 3.3 ABCB1

ABCB1, also known as P-glycoprotein (P-gp) and Multi Drug Resistance Protein (Mdr-1), functions together with low density lipoprotein receptor-related protein 1 (LRP1) to transport Aβ from the brain via the BBB (Hartz et al., 2010; ElAli and Rivest, 2013). It is encoded by the *ABCB1* gene in humans and by the *Abcb1a* and *Abcb1b* genes in rodents (Wang et al., 2016). ABCB1 has a wide range of substrates (Zhou, 2008) and is an important “gatekeeper” of the brain and other organs because of its role in blocking the entrance of many xenobiotics (foreign substances), including some chemotherapeutic agents, into these organs (Wang et al., 2016). It may also restrict entrance of Aβ into the brain (Candela et al., 2010). ABCB1 expression in the brain has been reported in endothelial cells, pericytes, microglia, and astrocytes (Sita et al., 2017), and it was reported on choroid plexus by Møllgård et al., 2017. The expression of ABCB1 on human brain endothelium has been reported to decrease, and to be inversely associated with cerebral Aβ levels, in the BBB during normal aging (Vogelgesang et al., 2002; Toornvliet et al., 2006; Bartels et al., 2009) and in AD (Wijesuriya et al., 2010; Deo et al., 2014; Chiu et al., 2015). Similar findings for ABCB1 expression and/or activity have been reported in animal models (Rosati et al., 2003; Hartz et al., 2010; Pekcec et al., 2011; Mehta et al., 2013; Zoufal et al., 2020a,b). However, opposite age-related results for ABCB1 expression in the BCSFB, i.e., an increase in its expression, were reported in male Brown-Norway/Fischer rats (Pascale et al., 2011). In the APP transgenic mouse model of AD, ABCB1 expression and transport activity at the BBB decreased prior to the development of cognitive deficits (Hartz et al., 2010).

The mechanisms which regulate ABCB1 expression are not well understood (Lim et al., 2008; Do et al., 2016). Schulz et al. (2023), reviewing pathways controlling ABCB1 and ABCG2 at the BBB (the transporters were discussed together in the review by Schulz et al. (2023) because they both restrict entrance of drugs to the brain), listed nuclear receptors [including corticoid receptors, retinoid X receptor (RXR), pregnane X receptor (PXR), constitutive androstane receptor, peroxisome proliferator-activated receptor (PPAR), and thyroid receptors], inflammation [nuclear factor-kappa B (NF-κB), Wnt/β-catenin signaling, TNFα, prostaglandins, and other cytokines], oxidative stress, receptor tyrosine kinases, and growth factor signaling. Vogelgesang et al. (2004), studying the relationship between ABCB1 and Aβ in human brain specimens with cerebral amyloid angiopathy (CAA), reported biphasic regulation: when Aβ was initially deposited in arterioles where ABCB1 expression was low, ABCB1 expression was upregulated in capillaries, but ABCB1 expression then decreased as Aβ accumulated in capillaries. A more recent study involving parietal cortex microvessels (Bourassa et al., 2019) found decreased ABCB1 expression in AD patients compared to its expression in clinically normal subjects and individuals with MCI. The concentration of ABCB1 in microvessels in AD patients was inversely correlated with vascular Aβ40 levels and positively correlated with measures of cognition and memory. The decrease in ABCB1 expression in AD brain may be due to increased cerebral Aβ burden (Brenn et al., 2011; Kania et al., 2011). Mechanisms suggested to account for decreased cerebral ABCB1 expression in AD include activation of the receptor for advanced glycation endproducts (RAGE) in conjunction with NF-κB signaling (Park et al., 2014), and systemic inflammation (Hartz et al., 2006; Salkeni et al., 2009; Erickson et al., 2012), which is present in some AD patients (Holmes and Butchart, 2011). The ubiquitin-proteasome system (UPS) is involved in regulation of ABCB1 expression and activity on the BBB (Loo and Clarke, 1998; Zhang Y. et al., 2004; Hartz et al., 2016), and Aβ-induced ubiquitination of ABCB1 has also been implicated in decreased cerebral expression of ABCB1 in AD (Akkaya et al., 2015; Hartz et al., 2016, 2018). Interestingly, although proteasomal clearance of ubiquitinated proteins has been reported to be decreased in AD brain (Perry et al., 1987; Keck et al., 2003), UPS activity may be increased on the AD BBB (Akkaya et al., 2015; Hartz et al., 2016, 2018).

Because of the expression of ABCB1 on the luminal surface of the BBB, it does not have direct access to Aβ in interstitial fluid, so the mechanism by which it mediates the efflux of Aβ is unclear. Storck et al. (2018) proposed that the Aβ efflux-promoting activity of ABCB1 may be functionally linked to that of LRP1 via phosphatidylinositol binding clathrin assembly protein (PICALM), whose genetic polymorphisms have been identified as risk factors for LOAD (Harold et al., 2009; Xu et al., 2015; Ando et al., 2022). They suggested that transfer of Aβ from LRP1 to ABCB1 may occur in Rab11-positive sorting endosomes, where both proteins are present.

Approaches that have been used to increase ABCB1 expression in experimental systems are shown in Table 3. Although one of the approaches listed is prevention of ABCB1 ubiquitination, effective therapeutic targeting of the UPS is problematic. As discussed by Hartz et al. (2018), designing of small molecule inhibitors for ubiquitination-related enzymes is difficult, and restricting the inhibition of ubiquitination to specific tissues is challenging.

**TABLE 3:** Experimental approaches for increasing ABCB1 expression.

Experimental approach	References
β-catenin signaling^a^	Lim et al., 2008
Colupulone analogs^b^	Bharate et al., 2015
Exosomes^c^	Pan et al., 2020
Ketone bodies	Versele et al., 2020
NMDA receptor agonists	Bauer et al., 2008
Nocodazole^d^	Ding et al., 2021
Oleocanthal	Abuznait et al., 2013
Pirenzepine^e^	Paganetti et al., 2014
Prevention of ABCB1 ubiquitination	Hartz et al., 2018
PXR agonists^f^	Hartz et al., 2010; Wolf et al., 2012; Lemmen et al., 2013; Zoufal et al., 2020b
TGF-β1	Baello et al., 2014
Vitamin D receptor activation	Durk et al., 2012

^a^β-catenin signaling was activated by inhibitors of glycogen synthase kinase-3β in primary rat brain endothelial cells and immortalized human brain endothelial cells (Lim et al., 2008).

^b^Colupulone analogs are PXR activators (Bharate et al., 2015).

^c^ABCB1-expressing human brain microvascular endothelial cell-derived exosomes were transplanted into a transgenic mouse model of AD by Pan et al. (2020).

^d^Nocodazole is a microtubule inhibitor which prevented ABCB1 internalization and subsequent degradation by the ubiquitin–proteasome system in Tg2576 mice (Ding et al., 2021).

^e^Administration of Pirenzepine, a selective M1 receptor antagonist, lowered cerebral Aβ in AβPPPS1, hAβPPSL, and AβPP/PS1 transgenic mice (Paganetti et al., 2014).

^f^PXR agonists include hyperforin, an active ingredient in St. John’s wort (Lemmen et al., 2013). Zoufal et al. (2020b) administered the rodent PXR activator 5-pregnen-3β-ol-20-one-16α-carbonitrile to APP/PS1-21 mice to activate cerebral ABCB1. ABCB1, ATP-binding cassette sub-family B member 1; NMDA, N-methyl-D-aspartate; PXR, pregnane X receptor; TGF-β1, tumor growth factor β1.

### 3.4 ABCC1

ABCC1 promotes the efflux of xenobiotic agents (including many chemotherapeutic agents and antibiotics), organic anions, and many compounds including the anti-oxidant glutathione (Wolf et al., 2012; Słomka et al., 2015). Its role as an export protein in AD has been studied mainly by Pahnke and colleagues (Krohn et al., 2011, 2015; Hofrichter et al., 2013; Schumacher et al., 2017; Zoufal et al., 2020c). Sultana and Butterfield (Sultana and Butterfield, 2004) suggested that it may play a neuroprotective role in AD by exporting glutathione-conjugated 4-hydroxy-2-trans-nonenal (HNE), a membrane lipid peroxidation product. HNE can be detoxified via its conjugation to the antioxidant glutathione. This activity is reduced in the AD brain (Lovell et al., 1998; Abuznait and Kaddoumi, 2012). A recent report suggested that in addition to its exporting of Aβ, ABCC1 may also lower Aβ levels by increasing the ratio of α- to β-secretase cleavage of APP (Jepsen et al., 2021).

ABCC1 is expressed in the CNS in many cell types including astrocytes, microglia, neurons, capillary endothelial cells, pericytes, neural stem and progenitor cells, and choroid plexus cells (Dallas et al., 2006; Bernstein et al., 2014; Pahnke et al., 2014; Matsumoto et al., 2015; Møllgård et al., 2017; Schumacher et al., 2017; Zoufal et al., 2020c). Its regulation was reported to be controlled by the transmembrane receptor protein Notch1 as well as transcription factors including GC (Guanine and Cytosine) elements and E-box elements (Cho et al., 2011). ABCC1 was reported to be upregulated *in vitro* in astrocytes following exposure to monomeric Aβ, resulting in increased release of glutathione; however, prolonged exposure to aggregated Aβ decreased ABCC1 activity (Ye et al., 2015). In the same study, brain ABCC1 transport activity was reported to be transiently increased in the 5×FAD mouse model of AD, with a subsequent decrease to below control levels. A study in APP/PS1 mice in which brain ABCC1 transport activity was measured at a single time point (170 days) found an increase in activity (Zoufal et al., 2020c). Although the cells responsible for this increase were not identified, it was suggested that an increase in ABCC1 activity in astrocytes, with a concomitant increase in their release of glutathione, may have been an effort to protect adjacent neurons from oxidative stress. Knockout of *ABCC1* in APP/PS1 mice with a different genetic background resulted in increased brain concentrations of aggregated Aβ40 and Aβ42 (Krohn et al., 2011). The increased cerebral Aβ42 levels in APP/PS1 mice could be prevented by activation of ABCC1, either by an extract of St. John’s wort (Hofrichter et al., 2013) or by the anti-emetic drug Thiethylperazine (Krohn et al., 2011). In the latter study, knockout of *ABCC1* in transgenic mice expressing the Dutch APP mutation [which causes CAA (Levy et al., 2006)] resulted in an increase in Aβ-immunoreactive cerebral vessels. Transport of Aβ42 in primary capillary endothelial cells from mouse brains in that study was reduced by approximately 60% by knockout of ABCC1, suggesting a major role for ABCC1 as an Aβ transporter.

The literature contains conflicting reports for the location of ABCC1 on the BBB [reviewed by Wolf et al. (2012)]. Some studies have found it on the luminal surface (Nies et al., 2004; Zhang Z. et al., 2004) while others have detected it on the abluminal surface (Soontornmalai et al., 2006; Roberts et al., 2008). Bernstein et al. (2014) concluded that ABCC1 was present on both luminal and abluminal membranes of brain capillary endothelial cells.

Experimental approaches to increase ABCC1 expression appear to have been limited, as indicated above, to administration of an extract of St. John’s wort (Hofrichter et al., 2013) and Thiethylperazine (Krohn et al., 2011). A phase 2 trial was initiated in November 2017 to investigate the safety and efficacy of Thiethylperazine in subjects with early onset AD (ClinicalTrials.gov Identifier: NCT03417986); although it was completed in 2021, results have apparently not been published. A potential drawback to the use of Thiethylperazine for treatment of AD is that although it promotes ABCC1-mediated transport activity, it inhibits the transport activity of ABCB1 (Wesołowska et al., 2009).

### 3.5 ABCC5

ABCC5 has been studied mainly because of its role in chemotherapeutic drug resistance. It was initially known as multidrug resistance protein 5 (MRP5) (Wijnholds et al., 2000). ABCC5 reduces access of some chemotherapeutic agents to tumors because of its activity as an efflux transporter of nucleoside/nucleotide analogs (Sampath et al., 2002; Ritter et al., 2005; Fukuda and Schuetz, 2012; Zhang et al., 2018). Another member of the ABC family, ABCC4 (MRP4), plays a similar role (Adachi et al., 2002). ABCC4 and ABCC5 are also efflux transporters for the second messengers 3′,5′-cyclic adenosine monophosphate (cAMP) and 3′,5′-cyclic guanosine monophosphate (cGMP) (Sampath et al., 2002; Wielinga et al., 2003; Ritter et al., 2005). Efflux of cAMP and cGMP may be an alternative mechanism to control their intracellular levels in addition to their degradation by phosphodiesterases (Meyer Zu Schwabedissen et al., 2005). ABCC5 also transports glutamate analogs including the excitotoxins N-methyl-D-aspartic acid and kainic acid (Jansen et al., 2015). ABCC5 expression has been detected in a range of tissues (Stojic et al., 2007); in the CNS it has been reported on the BBB and in neurons, astrocytes, microglia, and pericytes (Hirrlinger et al., 2002; Berezowski et al., 2004; Dallas et al., 2004; Warren et al., 2009; Jansen et al., 2015), as well as on the choroid plexus (Liddelow et al., 2012; Strazielle and Ghersi-Egea, 2015). ABCC5 expression was also detected on the hCMEC/D3 cell line (Carl et al., 2010), an immortalized human endothelial cell line suggested to be an *in vitro* model for the human BBB (Weksler et al., 2005). Comparison of mRNA levels of seven ABC transporters in human brain found the highest levels for ABCC5 and ABCG2 (Dutheil et al., 2009).

A PubMed search of “ABCC5 and Abeta clearance” found only one study. ABCC5 was reported to mediate transport of Aβ42 out of primary porcine brain endothelial cells (PBECs) (Shubbar and Penny, 2020). Although no reports were found of alterations of ABCC5 expression or activity in AD brain, the possibility that the ability of ABCC5 to prevent entrance of blood-borne Aβ into the brain may be impaired in AD was suggested by a study in which ABCC5 transporter activity was decreased after exposure of PBECs to Aβ42 (Shubbar and Penny, 2018).

ABCC5 expression was increased in CNE2 cells, an epithelial cell line (Sizhong et al., 1983), by increasing the expression of forkhead box M1, a cell growth-specific transcription factor (Hou et al., 2017). In another study, the anti-inflammatory agent dexamethasone increased ABCC5 expression and activity in primary PEBC cultures (Ho et al., 2023). Human chorionic gonadotropin was reported to regulate ABCC5 expression in human trophoblasts (Meyer Zu Schwabedissen et al., 2005).

### 3.6 ABCG1

ABCG1 is expressed in multiple organs (Nakamura et al., 2004); in the brain, it is present in neurons, astrocytes, and oligodendrocytes (Abildayeva et al., 2006; Kim et al., 2007; Tansley et al., 2007; Tarr and Edwards, 2008) and Liddelow et al. (2012) detected it on mouse choroid plexus. Optimal removal of cholesterol from cells is thought to require coordinated activity of ABCA1 and ABCG1 (Vaughan and Oram, 2005; Gelissen et al., 2006; Jasmin et al., 2014). ABCG1 transfers cholesterol to lipid-containing particles such as high-density lipoprotein (HDL), phospholipid vesicles, and lipidated apoE disks (Nakamura et al., 2004; Baldán et al., 2006; Kim et al., 2008; Tarr et al., 2009; Yu et al., 2010) whereas ABCA1 promotes the transfer of cholesterol and phospholipids to lipid-free (or lipid-poor) apolipoproteins (Oram and Heinecke, 2005; Prinz, 2007). In the brain, both transporters promote efflux of cholesterol from neurons to apoE (Alrosan et al., 2019). The expression of ABCG1, like ABCA1, is regulated by LXRs (Abildayeva et al., 2006; Cao et al., 2007; Burgess et al., 2008). A synthetic LXR activator, T0901317, upregulated expression of ABCG1, ABCA1, and apoE in the APP/PS1 transgenic mouse model of AD. Memory functions were improved but cortical and hippocampal SP counts were unchanged (Vanmierlo et al., 2011). Other regulators of ABCG1 include peroxisome proliferator-activated receptor (PPAR)-delta activators such as pioglitazone (Cocks et al., 2010), cellular sterol levels, and acute permeability barrier disruption (Jiang et al., 2006, 2010). Bexarotene was found to increase the expression of ABCG1 by activating RXR/LXR and RXR/PPAR heterodimers (Cramer et al., 2012; Zhao et al., 2014; Ren et al., 2019). Other RXR agonists have also been reported to increase ABCG1 expression (Boehm et al., 1995; Jung et al., 2010; Chen et al., 2011; Okabe et al., 2013; Sun et al., 2015). Although expression of ABCG1 has been reported in *in vitro* BBB models (Gosselet et al., 2009; Kober et al., 2017) and in the rat choroid plexus at the BCSFB (Fujiyoshi et al., 2007), ABCG1 does not appear to directly promote Aβ efflux from the brain. Conflicting results have been published regarding its effects on Aβ. Burgess et al. (2008) found that overexpression of ABCG1 in PDAPP mice did not change cerebral Aβ levels, similar to results in APP/PS1 mice in the study with synthetic LXR activator T0901317 discussed above (Vanmierlo et al., 2011). Kim et al. (2007) reported that expressing ABCG1 in APP-expressing Chinese hamster ovary cells reduced Aβ production, although Aβ’s clearance was unchanged, and Sano et al. (2016) found that expressing ABCG1 in human embryonic kidney cells containing the Swedish APP mutation decreased secretion of Aβ by these cells due to suppression of γ-secretase, an activity that was independent of ABCG1’s effects on lipid efflux. Kim et al. (2009) also reported that 27-hydroxycholesterol, which upregulated ABCG1, ABCA1, and apoE expression in primary human neuronal cultures, reduced Aβ in culture supernatants. [Conversely, (Prasanthi et al., 2009) was unable to detect upregulation of ABCG1 or ABCA1 by 27-hydroxycholesterol in SH-SY5Y cells, although cellular Aβ42 concentrations were increased; another oxidized cholesterol derivative, 24-hydroxycholesterol, did increase expression of ABCG1 and ABCA1, but this did not change cellular Aβ42 levels.] In contrast to studies which found upregulation of ABCG1 to be associated with decreased Aβ levels, other studies have found that ABCG1 upregulation increased Aβ levels. Tansley et al. (2007) reported that expressing ABCG1 in HEK cells containing the Swedish APP mutation increased Aβ production, and suggested that ABCG1 may have promoted APP processing by both amyloidogenic and nonamyloidogenic pathways. Dafnis et al. (2018) similarly found that expressing ABCG1 in SK-N-SH cells resulted in a moderate increase in Aβ production, which was suggested to be due to increased β-secretase activity.

In AD patients, the cholesterol efflux activity of cerebrospinal fluid (CSF), which is mediated by both ABCG1 and ABCA1 and promotes transfer of cholesterol from astrocytes to neurons, was reported to be reduced (Marchi et al., 2019). Cholesterol is necessary for optimal neuronal development and function (Dietschy and Turley, 2004; Hayashi et al., 2004; Göritz et al., 2007; Valenza et al., 2015). Because it does not cross the BBB (Vitali et al., 2014), neurons in the brain must be supplied by locally produced cholesterol. Adult neurons lose the ability to synthesize cholesterol (Dietschy and Turley, 2004) and obtain it from glial cells (Mauch et al., 2001; Pfrieger and Ungerer, 2011; Marchi et al., 2019). Significant associations between *ABCG1* single nucleotide polymorphisms (SNPs) and AD were detected in some European populations but not others (Wollmer et al., 2007).

### 3.7 ABCG2

ABCG2, also known as Breast Cancer Resistance Protein (BCRP) (Doyle et al., 1998), is expressed in many tissues including brain, liver, kidney, small intestine, mammary gland, and bone marrow (Zhou et al., 2001; Langmann et al., 2003; Zhang et al., 2003; Sarkadi et al., 2004; Blanco-Paniagua et al., 2021). It is present on the BBB on the luminal surface of microvessel endothelium (Cooray et al., 2002) where it co-localizes with ABCB1 Møllgård et al. (2017) and Wanek et al. (2020) reported its expression in human choroid plexus. Aβ is a substrate for ABCG2 (Xiong et al., 2009; Do et al., 2012; Shubbar and Penny, 2020). Whether ABCG2 transports Aβ out of the brain or prevents it from crossing the BBB into the brain is unclear. Evidence for ABCG2-mediated efflux of Aβ from the brain comes from a study which found that overexpressing human ABCG2 in immortalized rat brain endothelial cells increased abluminal-to-luminal transport of substrates (Zhang et al., 2003). Conversely, the possibility that ABCG2 may limit passage of Aβ from the BBB into the brain was suggested by the finding that inhibiting ABCG2 resulted in increased apical-to-basolateral transport of Aβ in endothelial cells (Tai et al., 2009). This was also suggested by another study which showed that although peripheral injection of Aβ40 resulted in greater brain accumulation of Aβ in ABCG2-knockout mice than in wild-type mice, the lack of ABCG2 did not appear to change the rate of Aβ elimination from the brain (Zhang et al., 2013). The influence of Aβ on ABCG2 expression and activity is unclear. While two studies found that exposure of human brain endothelial cells to Aβ did not influence ABCG2 expression (Xiong et al., 2009; Kania et al., 2011), another study found that ABCG2 efflux activity in porcine endothelial cells decreased following exposure of these cells to Aβ (Shubbar and Penny, 2018).

Conflicting reports have been published regarding changes in ABCG2 expression in transgenic mouse models of AD. Decreased numbers of ABCG2-immunoreactive brain microvessels were reported in APP/PS1 mice (Wanek et al., 2020) but upregulation of ABCG2 was reported in brain specimens from Tg-SwDI and 3×Tg mice (Xiong et al., 2009). Conflicting reports have also been published for changes in ABCG2 expression in brain specimens from individuals with AD and/or CAA (Xiong et al., 2009; Wijesuriya et al., 2010; Carrano et al., 2014; Kannan et al., 2017; Storelli et al., 2021) and associations between ABCG2 variants and the risk for developing AD (Cascorbi et al., 2013; Fehér et al., 2013).

Upregulation of ABCG2 has been suggested to protect neurons against reactive oxygen species and inflammatory cytokines by inhibiting nuclear factor kappa B activation (Shen et al., 2010) and, because ABCG2 is an efflux transporter of glutathione, by increasing extracellular glutathione concentrations (Brechbuhl et al., 2010). A role for ABCG2 in protecting against oxidative stress was also suggested by a study which found increased brain lipid/DNA oxidation in Tg-SwDI mice lacking ABCG2 (Zeng et al., 2012). The possibility that ABCG2 may reduce amyloidogenic processing of APP was suggested by a report that Aβ40 production decreased when ABCG2 was overexpressed in N2a-695 cells (Shen et al., 2010).

ABCG2 expression has been upregulated by exposure of human brain endothelial cells to conditioned medium from Aβ-activated microglia (Xiong et al., 2009), with similar findings in HEK293 cells after exposure to astrocyte-conditioned medium (Hori et al., 2004). Upregulation of ABCG2 in these studies may have been due to its activation by cytokines, because increased ABCG2 expression was detected in the human hCMEC/D3 cell line following exposure to inflammatory cytokines (Poller et al., 2010). Dexamethasone, stated above to increase ABCC5 expression and activity in porcine brain endothelial cells, also increased ABCG2 expression and activity in these cells (Ho et al., 2023). Exposure of brain endothelial cells to glutamate downregulated ABCG2 expression (Salvamoser et al., 2015). Other regulators of ABCG2 expression (discussed by Nakanishi and Ross, 2012) include hypoxia and hypoxia/oxidative-sensitive transcription factors (Krishnamurthy et al., 2004; Zeng et al., 2012), hormones (Yasuda et al., 2005; Wang et al., 2006), growth factors (Meyer zu Schwabedissen et al., 2006), gene amplification, epigenetic regulation (demethylation, histone acetylation, transcription factors including PPARγ, estrogen receptor alpha, progesterone receptor) (Mo and Zhang, 2012), and some chemotherapeutic agents, in agreement with the role of ABCG2 in multidrug resistance (Sukowati et al., 2012).

### 3.8 ABCG4

The structure of ABCG4 is similar to that of ABCG1. Like ABCG1 it promotes cholesterol synthesis (Tarr and Edwards, 2008) and efflux of cholesterol (as well as desmosterol, an intermediate in cholesterol synthesis) from cells to HDL but not to lipid-poor apolipoproteins (Wang et al., 2004; Vaughan and Oram, 2005; Kim et al., 2008). ABCG4 and ABCG1 have been suggested to play complementary roles in brain cholesterol homeostasis (Sun et al., 2014). ABCG4 may not be involved in the process by which adult neurons in the brain obtain cholesterol via efflux from glial cells (Abildayeva et al., 2006).

ABCG4 is expressed mainly in brain and eye (Annilo et al., 2001; Oldfield et al., 2002; Tarr and Edwards, 2008) although it has also been found in spleen, bone marrow, testes, skin and thymus (Yoshikawa et al., 2002; Wang et al., 2008). In the brain it is present primarily on glial cells and neurons (Wang et al., 2008; Sano et al., 2016). It was not detected in an *in vitro* model of the pig BBB (Kober et al., 2017) but was found on the mouse BBB, where it promoted efflux of both Aβ and desmosterol; interestingly, desmosterol and Aβ appeared to compete for ABCG4-mediated efflux (Dodacki et al., 2017). These findings followed an earlier report from the same investigators that ABCG4 mediated efflux of Aβ40 from HEK293 cells (Do et al., 2012). ABCG4 was also reported to be present on the choroid plexus, suggesting that it may promote efflux of Aβ out of the brain via the BCSFB (Fujiyoshi et al., 2007; Liddelow et al., 2012; Matsumoto et al., 2015). Both ABCG1 and ABCG4 inhibited γ-secretase activity in HEK 293 cells expressing the Swedish APP mutation (Sano et al., 2016), so this may be an additional mechanism by which they lower Aβ levels. ABCG4 expression was reported to be increased in plaque-associated microglia in AD brain (Uehara et al., 2008) and in brain microvessels from 3-month-old 3×Tg-AD mice (Do et al., 2016). Whether its expression in neurons and astrocytes changes during progression of AD is unknown.

Some but not all studies have found ABCG4 expression to be induced by LXRs (Engel et al., 2001; Abildayeva et al., 2006; Tarr and Edwards, 2008; Wang et al., 2008; Graham, 2015; Yang et al., 2021). Post-translational regulation of ABCG4 has been suggested to be related to its ubiquitination (Alrosan et al., 2019).

## 4 Discussion

The ABC transporters which influence Aβ levels in the brain have different mechanisms of action, some of which involve lipid metabolism (ABCA1, ABCA7, ABCG1, ABCG4). The expression of some of these transporters is decreased in AD brain and/or transgenic mouse models of AD (ABCB1, ABCG1) while others are increased (ABCA7, ABCC1, ABCG4; conflicting reports for ABCG2). If the expression and/or activity of the ABC transporters which promote efflux of Aβ from the brain or reduce its entrance into the brain could be increased therapeutically in AD patients, this might lower brain levels of soluble Aβ (and by doing so, perhaps the aggregation of Aβ to form SPs would be reduced). Whether such an approach used as “stand-alone therapy” would slow AD’s clinical progression is unknown. As indicated in the Introduction, efforts to slow AD progression by lowering brain Aβ levels failed in large-scale clinical trials until the recent findings with Lecanemab and Donanemab, and although the effects of these monoclonal antibodies on AD progression were statistically significant in their respective phase 3 trials, they were modest. Therefore even if cerebral expression of the ABC transporters discussed above could be increased therapeutically, it is unclear if this would slow AD’s neuropathological or clinical progression. The few studies in which expression of the relevant ABC transporters has been experimentally increased have had inconsistent results. Improvement of cognitive and/or memory impairments, or lowering of brain Aβ, was reported in transgenic mouse models of AD after treatment with ABCB1-upregulating exosomes (Pan et al., 2020), and the M1 receptor antagonist Pirenzepine, which upregulates ABCB1 (Paganetti et al., 2014); however, administration of Bexarotene to transgenic mouse models of AD produced conflicting results, overexpressing of ABCG1 in PDAPP mice did not change brain Aβ levels (Burgess et al., 2008) and treatment of 3×TgAD mice with Dexamethasone, which upregulates ABCC5 and ABCG2, exacerbated Aβ pathology (Green et al., 2006). Possible deleterious side effects would also be a concern with therapeutic increasing of the expression of the Aβ-regulating ABC transporters; as discussed above, treatment with Bexarotene and other LXR agonists can increase plasma triglycerides (Koldamova et al., 2010), and Thiethylperazine inhibits the transport activity of ABCB1 (Wesołowska et al., 2009) although it promotes ABCC1-mediated transport activity. *In vitro* findings suggesting that upregulation of ABCG1 may increased Aβ levels by increasing β-secretase cleavage of APP (Tansley et al., 2007; Dafnis et al., 2018) would also be a concern.

Literature searches were performed to identify human clinical applications for the experimental approaches which have been used to increase ABC transporter expression. These applications are shown in Tables 4–9. The highest number of human applications were found for approaches which increase the expression of ABCA1 (Table 4), ABCB1 (Table 5), ABCG1 (Table 8), and ABCG2 (Table 9). Clinical applications which would increase ABCC1 expression appear to have been limited to St. John’s wort and Thiethylperazine, and only *in vitro* studies were found for ABCG4 (Wang et al., 2004; Vaughan and Oram, 2006; Sano et al., 2016). Therapeutic interventions with relatively large numbers of clinical applications include Bexarotene (Table 4), exosomes, PXR agonists, ketone bodies, N-methyl-D-aspartate receptor agonists [which would be contraindicated in AD, because excitotoxicity has been implicated in AD-associated neurotoxicity (Wang and Reddy, 2017)], Pirenzepine (Table 5), Oleocanthal/extra virgin olive oil (Tables 4, 5, 8), Dexamethasone (Tables 7, 9), intermittent hypoxia, Progesterone, Epidermal Growth Factor, and Doxorubicin (Table 9).

**TABLE 4:** Clinical trials and clinical applications of experimental approaches to increase ABCA1 expression.

Experimental approach	Clinical trials and applications
Bexarotene	AD (NCT01782742), schizophrenia (NCT00141947, NCT00535574), Cushing’s disease (NCT00845351), psoriasis (NCT00151008), metastatic breast cancer (NCT00003752), acute myeloid leukemia (NCT00316030, NCT00615784), cutaneous T-cell lymphoma (NCT00178841, NCT05296304), breast cancer prevention (NCT00055991), aerodigestive tract cancer (NCT01116622), stage I-II lung cancer (NCT00125372), alopecia areata (NCT00063076), cutaneous T-cell non-Hodgkin lymphoma (NCT00660231), AIDS-related Kaposi’s sarcoma (NCT00002212), stage III/IV non-small cell lung cancer (NCT00514293)
LXR agonists	Atherosclerosis (Hong and Tontonoz, 2014)
PPAR-α and PPAR-γ activation	Dyslipidemia (Cheang et al., 2015), diabetes (reduction of cardiovascular events) (Lincoff et al., 2007; Asztalos et al., 2008); rheumatoid arthritis (Marder et al., 2013)
Retinoic acid receptor agonists	Emphysema (Stolk et al., 2012), advanced cancer (Miller et al., 1996; Soignet et al., 2000)
Verapamil	Cardiac arrhythmias, angina, hypertension (Mayo Clinic, 2023b)
Extra virgin olive oil^a^	Increasing of HDL-mediated cholesterol efflux (Helal et al., 2013)

^a^Consumption of extra virgin olive oil for 12 weeks by healthy volunteers increased macrophage expression of ABCA1 (Helal et al., 2013).

Clinical trials are identified by ClinicalTrials.gov identifier (NCT number). The effects of Bexarotene, a retinoid X receptor (RXR) activator, have been investigated in a large number of clinical trials. ABCA1, ATP binding cassette subfamily A member 1; PPAR-α, peroxisome proliferator-activated receptor-alpha; PPAR-γ, peroxisome proliferator-activated receptor-gamma.

**TABLE 5:** Clinical trials and clinical applications of experimental approaches to increase ABCB1 expression.

Experimental approach	Clinical trials and applications
β-catenin signaling^a^	Osteoporosis (Wu et al., 2023), mood disorders (Bonnet et al., 2021)
Colupulone analogs	Larvicidal agents (Makri et al., 2022)
Exosomes^b^	AD (NCT04388982), COVID19 (Mitrani et al., 2021); craniofacial neuralgia (NCT04202783), depression (NCT04202770), cutaneous wound healing (NCT02565264), multiple organ dysfunction after surgical repair of acute aortic dissection (NCT04356300), dry eye in chronic host vs. graft disease (NCT04213248), type 1 diabetes (NCT02138331), metastatic pancreatic cancer (NCT03608631), diagnostic and prognostic biomarker analysis (Liu et al., 2018; Qin et al., 2020; Chang et al., 2021)
Ketone bodies^c^	AD (NCT04701957), heart failure (NCT05768100), type 2 diabetes (NCT03657537, NCT04854330, NCT05155410), multiple sclerosis (NCT03740295), cardiogenic shock (NCT04642768), alcohol use disorder (NCT04616781), McArdle disease (NCT03945370), Parkinson’s disease and Lewy body dementia (NCT05778695), amyotrophic lateral sclerosis (ALS) (NCT02716662, NCT04820478), acute heart failure (NCT04442555, NCT05348460), eating disorders (NCT05507008), obesity (NCT03729934), polycystic ovary syndrome (NCT04163120), upper respiratory tract infections (NCT04019730), COVID-19 (NCT04573764), concussion (NCT04079907), aging (NCT06068803)
NMDA receptor agonists	Multidrug-resistant tuberculosis (Deshpande et al., 2018), urinary tract infections (Kugathasan et al., 2014), schizophrenia (Lakhan et al., 2013; NCT01474395, NCT00491569), nicotine dependency (Santa Ana et al., 2009), alcohol dependency (Watson et al., 2011), cocaine dependency (Price et al., 2009), major depression (NCT03062150), panic disorder (NCT00131339), post-traumatic stress disorder (NCT00215878, NCT00371176), depression (NCT01684163, NCT04721249), agoraphobia (NCT01928823), Parkinson’s disease (NCT00215904), obsessive-compulsive disorder (NCT02656342)
Nocodazole^d^	No human applications found
Oleocanthal/extra virgin olive oil^e^	MCI (NCT03362996, NCT03824197), type 2 diabetes (NCT04419948), chronic lymphocytic leukemia (NCT04215367), neurofibromatosis (NCT05363267), multiple sclerosis (NCT04787497), metabolic syndrome (NCT05282316); also see Table 3
Pirenzepine	Gastric and duodenal ulcers (Ishimori and Yamagata, 1982; Carmine and Brogden, 1985), reflux esophagitis (Niemelä et al., 1986), myopia (Tan et al., 2005; Siatkowski et al., 2008), hypersalivation (Bai et al., 2001), peripheral neuropathy (NCT04005287, NCT05488873, NCT04786340), HIV-associated polyneuropathy (NCT05005078)
Prevention of ABCB1 ubiquitination	No human applications found
PXR agonistsf: Rifampicin	AD (Molloy et al., 2013), primary biliary cirrhosis (Hoensch et al., 1985), drug-resistant Acinetobacter Baumannii (NCT03622918), tuberculosis (NCT01311505. NCT01986543), multiple system atrophy (NCT01287221), rhinoscleroma (NCT03326050), Staphylococcal infections (NCT02782078), non-small cell lung cancer (NCT05631678), endometriosis (NCT02975440), Parkinson’s disease (NCT04070495), osteoarticular infection (NCT02599493), HIV (NCT02832778), diabetes mellitus (NCT03063580), pulmonary arterial hypertension (NCT01251835), neoplasms (NCT01322438), antibiotic-associated diarrhea (NCT00182429), osteoarticular prosthetic infection (NCT00906048)
PXR agonists: St. John’s wort	Acne (NCT05073211), osteoarthritis (NCT05663996), nicotine dependence (NCT00405912), depression (NCT05477472, NCT00861978, NCT00066859, NCT04315597), phobic disorders (NCT00035412), obsessive-compulsive disorder (NCT00035438), Reynaud’s syndrome (NCT00351117), irritable bowel syndrome (NCT00587860), contraception (NCT00026013), anxiety disorders (NCT00118833, NCT00451516), perineal injury (NCT05164926), hot flashes (NCT00110136), attention deficit hyperactivity disorder (NCT00782080), peritoneal carcinomatosis (NCT02840331)
TGF-β1^g^	Residual burn-related wounds (NCT04235296)
Vitamin D receptor activation	Chronic kidney disease (Cozzolino and Malindretos, 2010; Yang et al., 2018), anemia associated with inflammation (NCT02876211), type 2 diabetes (NCT01393808, NCT00421733)

^a^β-catenin signaling for treatment of osteoporosis includes administration of Romozumab, a monoclonal antibody targeting sclerostin, an inhibitor of Wnt/β-catenin signaling (Wu et al., 2023). The effects of other anti-sclerostin monoclonal antibodies are being investigated in postmenopausal patients with decreased bone mass density (Bonnet et al., 2021). GSK-3β inhibitors are the most widely used Wnt/β-catenin activators. The GSK-3β inhibitor lithium chloride is used to treat mood disorders including bipolar disorder (Machado-Vieira et al., 2009) and major depression (Bauer et al., 2014).

^b^Clinical trials involving exosomes were reviewed by Aheget et al. (2020). A pilot study (NCT04388982) was performed to investigate safety and efficacy of treating AD patients (*n* = 9, divided into three treatment arms) with intranasally administered allogenic adipose mesenchymal stromal cell exosomes. Results were published by Xie et al. (2023). Cognitive functioning, measured by ADAS-Alzheimer’s Disease Assessment Scale-Cognitive section (ADAS-cog) and Montreal Cognitive Assessment (MoCA), was suggested to have improved in the medium-dose treatment group. A preparation derived from exosomes and extracellular vesicles of human amniotic fluid was used to treat three patients with severe COVID19. Improved clinical status, including respiratory function, was reported (Mitrani et al., 2021).

^c^A search of “ketone bodies” on ClinicalTrials.gov yielded 616 hits so only a partial list is shown. Notably, NCT04701957, “The Ketogenic Diet for Alzheimer’s Disease (CETOMA),” is evaluating the effects of a ketogenic diet on patients with early-stage AD. Patients will be followed for one year and changes in brain metabolism, cognition, quality of life, and activities of daily living functioning will be determined. The study is scheduled to be completed in March 2025.

^d^Nocodazole is an experimental anti-mitotic and anti-neoplastic drug which has been used to achieve cell cycle synchronization *in vitro* (Zieve et al., 1980; Blajeski et al., 2002; Khattab and Al-Karmalawy, 2021).

^e^Oleocanthal is a nonsteroidal anti-inflammatory agent found in extra virgin olive oil (Smith et al., 2005). Two clinical trials with extra virgin olive oil have been performed in subjects with MCI (NCT03362996, NCT03824197). Tzekaki et al. (2019) treated MCI patients with extra virgin olive oil for one year and compared serum fibrinolytic factors PAI-1 and a2-antiplasmin, as well as Aβ40, Aβ42, tau, and the oxidative stress marker malondialdehyde between these patients and non-treated MCI patients, AD patients, and healthy subjects. Post-treatment levels of both fibrinolytic factors and of tau and malondialdehyde decreased in the treatment group relative to the other groups, and the Aβ42/Aβ40 ratio in the treatment group was similar to that in the healthy subjects. In a later trial Kaddoumi et al. (2022) examined the effects of treating MCI patients with extra virgin olive oil or refined olive oil for six months. Treatment with extra virgin olive oil improved Clinical Dementia Rating scores and behavioral scores, while lowering BBB permeability and serum Aβ42/Aβ40 and p-tau/total tau ratios. Some of these effects were also found with refined olive oil. Other disorders in which the effects of oleocanthal have been investigated in clinical trials include type 2 diabetes (NCT04419948), chronic lymphocytic leukemia (NCT04215367), and neurofibromatosis (NCT05363267). Trials to examine the effects of extra virgin olive oil in multiple sclerosis (NCT04787497) and metabolic syndrome (NCT05282316) are recruiting.

^f^PXR agonists include Rifampicin and St. John’s wort. A partial list is shown for Rifampicin trials registered on ClinicalTrials.gov; the search result generated 369 hits. A search of St. John’s wort on ClinicalTrials.gov generated 37 hits. A one-year clinical trial in which patients with mild-to-moderate AD were treated with Rifampicin found no evidence for benefits in cognition or function (Molloy et al., 2013).

^g^Clinical trial NCT04235296, “Mesenchymal Stem Cell Conditioned Medium-derived Pleiotropic Factor in Treating Residual Burn Wound,” examined the effects of conditioned medium from mesenchymal stem cells on burn-related residual wounds. Mesenchymal stem cell conditioned medium contains TGF-β (Noh et al., 2016) and many other biological effectors (Ivanisova et al., 2023). Clinical trials are identified by ClinicalTrials.gov identifier (NCT number). ABCB1, ATP binding cassette subfamily B member 1; AD, Alzheimer’s disease; HIV, human immunodeficiency virus; MCI, mild cognitive impairment; NMDA, N-methyl-D-aspartate; PXR, pregnane X-receptor; TGF-β1, transforming growth factor beta 1.

**TABLE 6:** Clinical trials and clinical applications of experimental approaches to increase ABCC1 expression.

Experimental approach	Clinical trials and applications
St. John’s wort	See Table 5
Thiethylperazine^a^	AD (NCT03417986)

^a^NCT03417986 was a phase 2 trial investigating safety and efficacy of thiethylperazine in subjects with early onset AD. The status of the trial is listed on ClinicalTrials.gov as “Completed” (Actual Study Completion Date: October 22, 2021). The results of the trial have apparently not been published; no results were found with a PubMed search of “Thiethylperazine and Alzheimer’s.”

Clinical trials are identified by ClinicalTrials.gov identifier (NCT number). ABCCA1, ATP binding cassette subfamily C member 1; AD, Alzheimer’s disease.

**TABLE 7:** Clinical trials and clinical applications of experimental approaches to increase ABCC5 expression.

Experimental approach	Clinical trials and applications
Dexamethasone^a^	Multiple sclerosis; allergies; cerebral edema; inflammation; shock; COVID-19; asthma; atopic dermatitis; contact dermatitis; chemotherapy-induced nausea and vomiting; altitude sickness; tumor metastasis-related spinal cord compression (Teachey and Pui, 2019; Johnson et al., 2023); rheumatic diseases; pemphigus; severe erythema multiforme (Stevens-Johnson syndrome); exfoliative dermatitis; bullous dermatitis herpetiformis; severe seborrheic dermatitis; severe psoriasis; flare-ups ulcerative colitis, multiple sclerosis, myasthenia gravis (Medical News Today, 2022)
Increased expression of FOXM1^b^	No human applications found

^a^Dexamethasone is a glucocorticoid used clinically to prevent and reduce inflammation. Although inflammatory mechanisms are increased in AD brain (Kinney et al., 2018; Kiraly et al., 2023), clinical trials with anti-inflammatory agents found no evidence for slowing of AD clinical progression (Aisen et al., 2000, 2003). Chronic treatment with dexamethasone was found to damage hippocampal neurons *in vitro* (Zhang et al., 2017) and *in vivo* (Hu et al., 2016), and dexamethasone administration increased tau phosphorylation in 3×Tg and Tg2576 mice (Joshi et al., 2012, 2013). A later study with 3×Tg mice found that treatment with dexamethasone exacerbated both Aβ and tau pathology (Green et al., 2006).

^b^Increasing FOXM1 to increase ABCC5 expression in AD patients may be contraindicated, based on a study of FOXM1 staining in 236 patients with breast cancer (Ahn et al., 2015). Increased FOXM1 immunoreactivity correlated with adverse clinicopathological features including larger size of tumors, metastasis to lymph nodes, and advanced tumor stage. ABCC5, ATP binding cassette subfamily C member 5; AD, Alzheimer’s disease; COVID19, coronavirus disease 2019; FOXM1, forkhead box M1.

**TABLE 8:** Clinical trials and clinical applications of experimental approaches to increase ABCG1 expression.

Experimental approach	Clinical trials and applications
ABCG1 overexpression	No human applications found
Extra virgin olive oil^a^	See Oleocanthal/extra virgin olive oil, Table 4
LXR agonists	See Table 4
PPAR-δ activation	See Table 4
RXR activation (Bexarotene)	See Table 4

^a^Consumption of extra virgin olive oil for 12 weeks by healthy volunteers increased macrophage expression of ABCG1 (Helal et al., 2013).

The approaches, with the exception of ABCG1 overexpression, are the same as listed for ABCA1 in Table 4. ABCG1 ATP binding cassette subfamily G member 1; LXR, liver X receptor; PPAR-δ, Peroxisome proliferator-activated receptor-delta; RXR, retinoid X receptor.

**TABLE 9:** Clinical trials and clinical applications of experimental approaches to increase ABCG2 expression.

Experimental approach	Clinical trials and applications
**Inflammatory cytokines**	
IFN-γ	Drug-resistant tuberculosis, chronic granulomatous disease, osteopetrosis (Donnelly et al., 2009; Miller et al., 2009)
IL-2	Malignant melanoma, renal cell carcinoma (Chavez et al., 2009)
IL-7	Expansion of T cells in patients with cancer, HIV, or allogeneic transplantation (Sportès et al., 2009)
Dexamethasone	See Table 7 (ABCC5)
Hypoxia (intermittent)^a^	MCI (NCT05495087), enhancement of cognition in older adults (NCT03957213), sleep apnea (Gerst et al., 2001; Yokhana et al., 2012), chronic obstructive pulmonary disease (Serebrovskaya et al., 2003), enhancement of aerobic exercise performance (Fulco et al., 2000; Shatilo et al., 2008), systemic hypertension (Serebrovskaya et al., 2008), coronary artery disease (Korkushko et al., 2010), obesity (NCT02973438), prevention of acute hypoxia injury (during mountain climbing) (NCT05733338, NCT04725539)
Progesterone^b^	Contraception, maintenance of pregnancy, postmenopausal symptomatic therapy, secondary amenorrhea, abnormal uterine bleeding (Fedotcheva, 2021), prevention of endometrial hyperplasia (Apgar and Greenberg, 2000), assisted reproductive technology (luteal phase support during *in vitro* fertilization) (Nagy et al., 2021), termination of premature labor (Fitzpatrick and Good, 1999)
Other hormones (EGF)^c^	Enhancement of peripheral wound healing, treatment of gastrointestinal damage (Berlanga-Acosta et al., 2009), necrotizing enterocolitis, Zollinger–Ellison syndrome, gastrointestinal ulceration and congenital microvillus atrophy (Guglietta and Sullivan, 1995); Myogenic Temporomandibular Disorder (NCT06044974), diabetic foot ulcer (NCT02554851), burn wounds (NCT01553708), oral mucositis (NCT04995354)
Doxorubicin	Soft tissue and bone sarcomas and cancers of the breast, ovary, bladder, and thyroid; acute lymphoblastic leukemia, acute myeloblastic leukemia, Hodgkin lymphoma, small cell lung cancer (Johnson-Arbor and Dubey, 2023), mesothelioma (de Lima and Sørensen, 2015)

^a^Clinical uses of intermittent hypoxia were reviewed by Navarrete-Opazo and Mitchell (2014). Clinical trial NCT05495087 (sponsor: University of North Texas Health Science Center) is a phase I trial to examine safety and efficacy of intermittent hypoxia training for up to 12 weeks in subjects with MCI. The study was first posted 10 August 2022 and is currently in recruiting phase.

^b^ClinicalTrials.gov listed 1,954 studies for progesterone. A partial list containing the main clinical uses of progesterone is shown.

^c^A search of EGF on ClinicalTrials.gov yielded 977 hits. A partial list is shown.

Clinical trials are identified by ClinicalTrials.gov identifier (NCT number). EGF, epidermal growth factor; IFN-γ, interferon-γ; IL-2, interleukin-2; IL-7, interleukin-7; MCI, mild cognitive impairment.

Few clinical trials have investigated the effects of therapeutic approaches which increase the expression of ABC transporters in AD patients or individuals with MCI. A phase 2 trial with Bexarotene was performed in AD patients (Cummings et al., 2016); lowering of brain Aβ was found in apoE4 noncarriers but not in apoE4 carriers. A clinical trial with the PXR agonist Rifampicin found no benefits on cognition or functioning in AD patients (Molloy et al., 2013). A phase I/II study involving intranasal administration of mesenchymal stromal cell exosomes to AD patients concluded that this approach was safe, and cognitive functioning was suggested to have improved in the medium-dose treatment group (Xie et al., 2023).

Administration of extra virgin olive oil to subjects with MCI for one year decreased serum fibrinolytic factors, the Aβ1-42/Aβ1-40 ratio, and the oxidative stress marker malondialdehyde (Tzekaki et al., 2019), while another study found that six-month administration of extra virgin olive oil to individuals with MCI improved their clinical dementia rating and behavioral scores (Kaddoumi et al., 2022). Among studies investigating the effects of ketogenic diets or ketogenic supplementation in AD patients (reviewed by Lilamand et al., 2022), one study found no cognitive change (Henderson et al., 2020) while others reported improvements in activities of daily living and quality of life (Phillips et al., 2021) and cognitive functioning (Xu et al., 2020). Improved memory or cognition was also found in two studies with ketogenic diet-treated subjects with MCI (Fortier et al., 2020; Neth et al., 2020). As stated above, results have not been published from a phase 2 trial to investigate the safety and efficacy of Thiethylperazine in early onset AD. A trial to investigate the efficacy of intermittent hypoxia in patients with MCI (NCT05495087) is in the recruiting stage. Other approaches for increasing ABC transporter expression which might be worthwhile to evaluate in AD pilot studies include LXR agonists, retinoic acid receptor agonists, β-catenin signaling, Verapamil, and St. John’s wort.

With regard to additional future research directions: evidence for the ability of some of the ABC transporters discussed above to promote clearance of cerebral Aβ is based primarily on findings that knockout of these transporters decreased Aβ efflux or increased Aβ levels *in vitro* or in transgenic mouse models of AD. The effects of approaches to increase the expression of the relevant ABC transporters could be examined in these models. Studies could also be performed in mouse models of AD to determine if increasing the expression of Aβ-regulating ABC transporters would further slow the progression of AD-related neuropathology or cognitive deficits when combined with administration of Lecanemab or Donanemab; if encouraging findings are obtained, this could be examined further in an AD pilot study.

## 5 Conclusion

Eight members of the human ABC transporter superfamily have been suggested to participate in clearing of Aβ from the brain. Although these transporters promote Aβ clearance by different mechanisms, several of them do so as a consequence of their involvement in regulation of lipid metabolism, including promoting synthesis and efflux of cholesterol. Some of the transporters may contribute to lowering of cerebral Aβ indirectly, for example by increasing non-amyloidogenic cleavage of APP, increasing phagocytic clearance of Aβ, exerting neuroprotective effects (such as exporting neurotoxic lipid peroxidation products, inhibiting nuclear factor kappa B activation, and increasing extracellular glutathione), and preventing Aβ from entering the brain via the BBB. The expression of ABC transporters involved in cerebral Aβ clearance has been increased through many experimental approaches, some of which are commonly used to treat conditions unrelated to AD; however, few of these approaches have been investigated for efficacy in AD patients. The possibility that therapeutic upregulation of selected ABC transporters might slow AD progression should be further explored.

## Author contributions

DL: Conceptualization, Writing – original draft, Writing – review & editing.
